# Impulsive and Compulsive Buying Tendencies and Consumer Resistance to Digital Innovations: The Moderating Role of Perceived Threat of COVID-19

**DOI:** 10.3389/fpsyg.2022.912051

**Published:** 2022-06-02

**Authors:** Hung Xin Li, Komal Hassan, Haider Ali Malik, Marhana Mohamed Anuar, Tariq Iqbal Khan, Mohd Rafi Yaacob

**Affiliations:** ^1^Department of Logistics Management, Faculty of Operation Research, National Defence University, Taoyuan, Taiwan; ^2^Department of Home Economics, Faculty of Arts and Social Sciences, Lahore College for Women University, Lahore, Pakistan; ^3^FAST School of Management, National University of Computer and Emerging Sciences, Islamabad, Pakistan; ^4^Faculty of Business Economics and Social Development, Universiti Malaysia Terengganu, Kuala Nerus, Malaysia; ^5^Department of Management Sciences, The University of Haripur, Haripur, Pakistan; ^6^Faculty of Entrepreneurship and Business, Universiti Malaysia Kelantan, Kota Bharu, Malaysia

**Keywords:** impulsive buying tendency, compulsive buying tendency, consumer resistance, digital innovation, perceived threat of COVID-19

## Abstract

Based on the theory of reasoned action and innovation resistance theory, this study aims to explore the tendencies of consumer resistance to digital innovation and the moderating role of a perceived threat of coronavirus disease 2019 (COVID-19). Data were collected using a cross-sectional online survey of 1,000 consumers of fast-moving consumer goods (FMCGs) in Pakistan. The results revealed several significant relationships between tendencies (impulsive and compulsive) of consumer resistance to digital innovation and the perceived threat of COVID-19. This study brings several key insights for consumers of FMCG products from Pakistan, and many theoretical and practical implications and future research directions are suggested.

## Introduction

Digital innovation is a critical component of a company’s long-term survival and growth, and it has been referred to as the “vital part” of most businesses (Hosseini et al., 2016). “Consumer resistance,” which seems to have been overlooked in the research literature, is one of the key factors preventing technology acceptance. Even though the novel item may bring substantial advantages and enhanced functionality, studies have discovered that customers are often less than enthused about a variety of new items (Hosseini et al., 2016). It has been described as “Innovation resistance is the resistance offered by consumers to an innovation, either because it poses potential changes from a satisfactory status quo or because it conflicts with their belief structure” (Abbas et al., 2017). “It is critical to the success of innovation since it can stifle or postpone user acceptance. It has been identified as among the main causes of technology financial distress and a useful source of knowledge critical to the successful deployment and promotion of development” (Talwar et al., 2020). Consumer resistance has been a significant challenge for businesses, and it will be proved to be so in the coming years (Heidenreich and Kraemer, 2016). Experts claim that companies must first know the cause of production delays (Abbas et al., 2017). Fast-moving consumer goods (FMCGs) are products that have a short lifetime and are commonly designed for single and limited uses (Bocken et al., 2022). In the context of FMCGs, consumer resistance to digital innovation would delay the adoption of innovation among consumers. Hence, consumer resistance to innovation is an essential topic that academicians and researchers must explore to guarantee the rapid transmission and implementation of new technologies (Joachim et al., 2018; Sadeghi, 2020).

Research has been performed on impulsive buying, referred to as “unplanned buying” (Efendi et al., 2019). However, it is eventually defined as “an emotional experience in which the speed of behavior prevented thoughtful consideration of alternatives or repercussions” (Hubert et al., 2013). Impulsive buying is stated as a user’s purchase behavior that is not premeditated. According to impulsive buying behaviors, customers quickly want to buy when they are between an item and a cashier (Panagiotidou, 2021). Buyers and clients frequently make unplanned buying, with which brands and distributors are well aware of. As a result, they appeal to purchasers’ or customers’ spontaneous desires (Urquia et al., 2019; Panagiotidou, 2021). The features of impulsive buying are as follows; “(1) spontaneities, (2) strength, (3) compulsion, (4) intensity, (5) excitement, (6) stimulus, and (7) indifference will result” (Xiao and Nicholson, 2013). Until ultimately did Amos et al. (2014) describe impulsive buying as a restricted challenge policy, claiming that the search for information before assessments was reduced in the impulsive buying judgment procedure. Similarly, the widely praised idea of planned behavior is found on the premise that “human beings were usually quite reasonable and made systematic use of accessible information” (O’Brien and McKay, 2016).

Joireman et al. (2010) referred to compulsive buying as “chronic, repetitive purchases that become a primary response to negative events or feelings. The activity, while perhaps providing short-term positive rewards, becomes very difficult to stop and ultimately results in harmful consequences.” Some experts have investigated that compulsive buying might be thought of as a spectrum within several customers rather than a classification factor (“i.e., a consumer is a compulsive buyer only if his or her score on a clinical screener for compulsive buying surpasses a specific threshold”) (Joireman et al., 2010). The term “compulsive buying” relates to an uncontrollable and excessive need to explore and purchase items (Japutra and Song, 2020). It is described as irrational, obsessive, and pointless obsessions with purchasing products that correlate to bouts of uncontrollable purchasing of goods that the customer cannot earn or just does not require (Albon et al., 2018; Hamid et al., 2019; Lawrence and Elphinstone, 2021).

People with greater levels of distress have a proclivity to see situations as extremely dangerous, and they frequently have inadequate coping skills, personality, and estimates of prevalence, resulting in effective anxiety (Purba and Sitorus, 2017; Kroencke et al., 2020; Paredes et al., 2021). According to a study conducted during the coronavirus disease 2019 (COVID-19) epidemic, people with high anxiety had greater perceptions of the COVID-19 viral threat, which increased adverse effects (Haq et al., 2010; Kim, 2020). Individuals with higher degrees of perfectionism, marked by high main objective traits, may be more open to COVID-19 intervention strategies. In conclusion, this feature may boost as they are considering the impact of avoiding COVID-19 while reducing their perception of COVID-19 as a threat, leading to less anxiety (Kim et al., 2020; Ahn and Kwon, 2022). It is defined as “the situations that were difficult or troubling to the individual and were described by respondents in narrative form. Degree of threat was then measured by one item on which subjects indicated the degree of concern the threatening event had caused them” (Paredes et al., 2021). Past research indicated that when it comes to technology resistance or acceptance, consumers of FMCG products usually rely on their normative beliefs for the decision to adopt or resist (Armitage and Conner, 2001; Khan et al., 2018; Ahmad, 2021). Previous research has also explained that the perceived threat of COVID-19 may moderate the decision-making process and its motivators (Sivathanu, 2018; Liu et al., 2021; Paredes et al., 2021), thus providing a logical theoretical reason to expect similar threatening effects in the case of impulsive and compulsive buying tendencies of FMCG consumers in low-income societies.

Finally, the present study has been established on reasoned action and innovation resistance theory. It is based on “human beings were usually quite rational and made systematic use of accessible information” (Badgaiyan et al., 2016). It demonstrates the importance of norms in customer decisions, and studies on technology acceptance reveal that customers generally depend on their normative beliefs when deciding whether or not to use technologies (Armitage and Conner, 2001; Karim et al., 2021). The theory of innovation resistance is defined as “the behavior toward the adoption and usage of any innovation that results in maintaining the status quo and resisting any deviances from the current beliefs” (Kaur et al., 2020). This research deals with impulsive and compulsive buying tendencies and consumer resistance to digital innovations, and the moderating role of a perceived threat of COVID-19. Hence, these theories are the foundation for the proposed theoretical framework being empirically investigated in this research. The expected rise of the global FMCG market is estimated at around 15,361.8 billion in 2025 as per allied market research data (Panhwar et al., 2022). This research is conducted in the context of the Pakistani consumer market of FMCG consumers to tap the influence of impulsive and compulsive buying tendencies on consumer resistance to digital innovation. This context has several merits in conducting this study as the consumer resistance to digital innovation in low-income societies is an emerging theme of research (Mazhar et al., 2012; Qazi et al., 2014; Akram et al., 2022). This study is expected to bring several key policy insights to developing countries, thus providing empirical evidence from Pakistan’s low-income society which is expected to bridge the existing literature gap and advance the current body of knowledge.

As a result, this research aims to look into impulsive and compulsive buying tendencies, consumer resistance to digital innovations, and the moderating role of a perceived threat of COVID-19. The objectives of this study are as follows:

1. To examine the impact of impulsive and compulsive buying tendencies on consumer resistance to digital innovation.
2. To examine the moderating effect of perceived threat of COVID-19 on the relationship between impulsive buying tendency and consumer resistance to digital innovations.
3. To examine the moderating effect of perceived threat of COVID-19 on the relationship between compulsive buying tendency and consumer resistance to digital innovations.

## Literature Review

### Theoretical Framework and Hypothesis Development

The research focuses on impulsive and compulsive buying tendencies, consumer resistance to digital innovations, and the moderating role of a perceived threat of COVID-19. The present study has been related to the theory of reasoned action and innovation resistance theory. It is based on “human beings were usually quite rational and made systematic use of accessible information” (Badgaiyan et al., 2016). The theory of reasoned action permits innovative scholars and practitioners to examine the influence factors of both pro and con uptake considerations (Badgaiyan et al., 2016; Karunaratne et al., 2021). The theory of reasoned action relates to how impulsive and compulsive buying tendencies impact online shopping and, during COVID-19 which types of threats consumers face. The theory of innovation resistance is described as “the behavior toward the adoption and usage of any innovation that results in maintaining the status quo and resisting any deviances from the current beliefs” (Kaur et al., 2020). The hypothesis aids in the comprehension of consumers’ inhibition behavior. It is carried out in a manner arising from logical thought and judgment toward implementing technology due to the potential for change given about by modifications to established order and variances from the current religious ideology (Kaur et al., 2020). The theory of innovation resistance relates to consumer resistance to digital innovations. The respective institution and empirical objectives posed in this study are critical to combine various theories into a coherent framework connected to impulsive and compulsive buying tendencies and consumer resistance to digital innovations and moderating the role of perceived threat of COVID-19.

### Consumer Resistance to Digital Innovations

Consumer resistance to innovation can be discussed in terms of the adoption behavior of an innovation. Factors such as consumer awareness and resistance influence the adoption process of innovation (Claudy et al., 2015; Joachim et al., 2018). The literature highlights two types of resistances to innovation. First, passive innovation resistance results at the end of the adoption process in its early stage due to the rejection of innovation before its evaluation (Bagozzi and Lee, 1999; Pacheco et al., 2022). Second, consumers with high readiness for the mental effort evaluate innovation in the persuasion stage in which different product specifics are evaluated. Hence, consumers gather reasons to either adopt or reject an innovation that will lead to attitude formation (Kleijnen et al., 2009; Shahbaz et al., 2014). Positive attitude formation leads to active innovation acceptance; in contrast, negative attitude formation results in active innovation resistance.

### Impulsive Buying Tendency and Consumer Resistance to Digital Innovations

The value of spontaneous purchases is very well-understood in the retail industry. Several facets of impulsive buying intentions have been studied for decades by investigators. While some academics have looked at the function of internal elements such as purchasing behavior, purchasing delight, consumerism, psychology, and society in impulsive buying, everyone has attempted to uncover the influence of different factors on impulsive buying (Panagiotidou, 2021). It has been described as “the degree to which an individual is likely to make unintended, immediate, and unreflective purchases” (Amos et al., 2014). Similarly, impulsive buying is “consumer buying impulsivity” and combines expressive and intellectual objects to make higher-order characteristics (Urquia et al., 2019). Many studies utilize the impulsive buying tendency as a surrogate for rapid purchase behavior, which is incorrect. The first represents a durable consumer feature that provides desires or reasons for purchasing, whereas the latter shows impulsive buying behavior (Badgaiyan et al., 2016; Kakkar et al., 2022). Impulsive buying is classified as a customer’s purchase behavior that is not organized (Efendi et al., 2019). According to impulsive buying actions, customers have a quick want to purchase when they become between an item and a cashier (Waheed et al., 2013; Amos et al., 2014). Customers and clients frequently make impulsive purchases, with which brands and distributors are well aware of. As a result, they pique the attention of impulsive customers.

Thus, consumer resistance to digital innovation is “resistance to the innovation offered by the consumer toward an innovation, either because it possesses potential changes from a satisfactory status quo or because it conflicts with their beliefs structure” (Hong, 2020). A limited proportion of research on this topic concentrated on the function of resistance in the acceptance of products and goods (Laukkanen, 2016). From a social perspective, activity is determined as an unpleasant incentive form that arises whenever anyone who perceives his independence is challenged, resulting in an understanding and activity to regain the vulnerable ability. The main reasons for choosing consumer resistance to digital innovation include (a) such developments are beginning to transform people’s lives in ways (Laukkanen, 2016); (b) online goods and services enhancing social levels of innovation and yet are challenging to handle; and (c) smart technological breakthroughs have been flexible (Hong, 2020), shortening the invention lifetime of established advancements and confusing people in customers’ imaginations (Sivathanu, 2018). As a result, a large body of evidence demonstrates a direct link between impulsive buying and consumer resistance to digital innovations. Thus, the following hypothesis is suggested:

**H1:** Impulsive buying tendencies have a significant impact on consumer resistance to digital innovations.

### Compulsive Buying Tendency and Consumer Resistance to Digital Innovations

The term “compulsive buying” is defined as an uncontrollable and excessive need to explore and purchase items (Japutra and Song, 2020). Combined self-control and attention deficit behaviors are present in compulsive buying. It is stimulated by unstoppable or unmanageable impulses that result in serious behaviors, whereas indulgent compulsive buying creates anxiousness and overly emotional opinions that interfere with everyday life since many factors of a person’s experience revolve around the decisions made (Farooq et al., 2010; Tarka and Kukar-Kinney, 2022). He et al. (2018) defined compulsive buying as “a response to an uncontrollable drive or desire to obtain, use, or experience a feeling, substance, or activity that leads an individual to repetitively engage in a behavior that will ultimately cause harm to the individual and/or to others.” As a result, the impacted customer engages in actions that can provide a diversion and assist in diverting one’s attention away from their current concerns (Roberts et al., 2014). Compulsive buyers, however, may not gain immediately through their purchasing; rather, people feel a brief burst of satisfaction and joy throughout the purchasing. Customers who engage in compulsive buying can explore happy emotions while doing so (Joireman et al., 2010; Liu et al., 2022).

Consumers can deliberately fight innovations. When customers experience effective resistance, they do not choose a new service for a long period after evaluating unique ideas and when development has occurred (Abbas et al., 2017). These types of customer resistances to innovation result in various effects ranging from the backlash to different reactions such as customer complaints or changes in customer mindset (Hong, 2020). Customer resistance to innovation refers to “the action or process of innovating, or to a new method, product or idea, innovation overlaps design and creativity, requiring the application, implementation, and explanation of new ideas to deliver an intended result, new customer, new markets, bigger markets or competitive advantage” (Talwar et al., 2020). Some researchers suggest that compulsive buying tendencies and customer resistance to innovation have significant relations (Heidenreich and Kraemer, 2016; Laukkanen, 2016; Hong, 2020; Shi et al., 2020; Talwar et al., 2020).

Hence, it is hypothesized that

**H2:** Compulsive buying tendencies have a significant impact on consumer resistance to digital innovations.

### The Moderating Role of Perceived Threat of COVID-19

**FIGURE 1:**
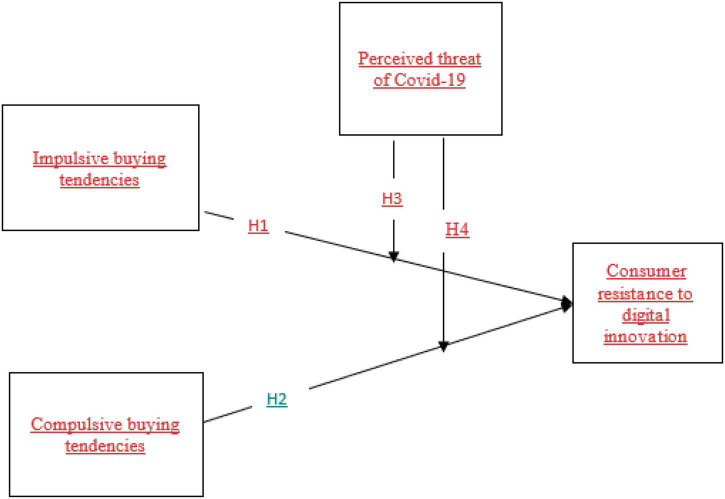
Research model.

The ongoing “coronavirus pandemic” has put the world’s trade under exceptional strain. Due to the increased potential of chemical exposure on-premises, the industries are proved to be the ones affected by the COVID-19 infection (Shin and Kang, 2020). The elevated risk factor is the cause of traveling apprehension after the COVID-19 epidemic. Scientific advancements, environmental disasters, and population concerns all pose challenges globally. On either side, the “coronavirus (COVID-19) pandemic” has appeared as the decade’s most critical problem. It has drastically altered people’s careers and significantly impacted their physical, personal, and living circumstances (Khan et al., 2021). Consumers’ anxiety about COVID-19 grew significantly due to unpredictability, job instability, economic stress, and federal health policy. The administration’s unprecedented steps to contain the spread sparked fear and exacerbated depression or anxiety. Consumers were immediately impacted by the change in market operations (Paredes et al., 2021). As a result, insights from academic studies on reducing resistance are critical to keeping up with digitalization. However, prior researchers have noticed that this field has stayed under-represented, with insufficient study devices, digital platforms, merging goods, smartphone e-commerce, and other technologies (Sivathanu, 2018; Paredes et al., 2021). It demonstrates the importance of norms in customer decisions, and studies on technology acceptance reveal that customers generally depend on their normative beliefs when deciding whether or not to use technologies (Armitage and Conner, 2001; Ahmad, 2021). The study of customer resistance to more technologies in the context, depending on innovations and customer behavior, can promote innovation, investigation, and a new group of details about customers’ behavior against newer technology (O’Brien and McKay, 2016). Finally, assemblers would be more able to forecast customers’ reactions/interactions with new items, allowing them to reduce consumer resistance. It is defined as “to describe, explain, and predict how consumers respond to innovation” (Talwar et al., 2020). Some researchers suggest that the perceived threat of COVID-19 shows a moderating effect (Sivathanu, 2018; Urquia et al., 2019; Liu et al., 2021; Paredes et al., 2021). As a result, a large body of evidence demonstrates that the perceived threat of COVID-19 has a significant impact on consumer resistance to digital innovations. The theoretical framework of this study is presented in Figure 1.

As a result, individuals worldwide have been advised to remain at home and participate in “physical or social separation.” The COVID-19 pandemic, like past infectious disease outbreaks, caused consumer resistance which led to large compulsive and impulsive buying (Efendi et al., 2019). Consumers with a higher perceived threat are more likely to experience adverse effects on their conscious emotional well-being. People’s views of how COVID-19 may create an unpleasant result that may have harmful effects on their lives are linked to threats in the COVID-19 instance (Liu et al., 2021). Consumers have also been demonstrated to feel fear due to the imagined COVID-19 threat, which could be harmful to their personal well-being (Kim, 2020). The perceived threat of COVID-19 influences impulsive buying tendencies. Due to the corona virus, people stay in their homes and do not go outside. In impulsive buying, they have an immediate want to purchase when they become between an item and a cashier; according to impulsive buying actions, all activities stop and close due to the threat of COVID-19 (Badgaiyan et al., 2016). Generally, impulsive buying means unplanned buying, but in a pandemic situation, people do not go for shopping, that is why perceived threat effects on impulsive buying tendencies.

**H3:** Perceived threat of COVID-19 moderates the relationship between impulsive buying tendency and consumer resistance to digital innovations so that the relationship is weaker when the perceived threat of COVID-19 is high.

The perceived threat of COVID-19 influences compulsive buying tendencies. Compulsive buying means uncontrolled punchers and item buying. It is described as irrational, obsessive, and pointless obsessions with purchasing products that correlate to the bouts of uncontrollable purchasing of goods that the customer cannot earn or just do not require (He et al., 2018), that is why, perceived threats impact compulsive buying tendencies. Consumer resistance is also affected by the perceived threat of COVID-19 because it is a dangerous disease. Consumer resistance has been a significant challenge for businesses, and it will prove to be so in the coming years (Kaur et al., 2020). Hence, the perceived threat of COVID-19 plays a moderating role between impulsive buying tendency, compulsive buying tendency, and consumer resistance to digital innovations. Thus, it is hypothesized that

**H4:** The perceived threat of COVID-19 moderates the relationship between compulsive buying tendency and consumer resistance to digital innovations in such a way that the relationship is weaker when the perceived threat of COVID-19 is high.

## Research Methodology

The study’s theoretical framework was developed, and hypotheses were proposed based on a thorough review of the literature and the theory of reasoned action and innovation resistance theory.

### Participants and Procedure

One of the multinational FMCG company head offices located in Lahore, Pakistan, was contacted through the personal reference of one of the study authors. They were requested to provide the verified consumer data of emails collected through a lucky draw in a marketing campaign after providing a strict guarantee that these data will not be used other than research purpose and will not be provided to any third party at any research stage. They agreed to share the email addresses of consumers. To obtain formal authority to involve their employees in this inquiry, a letter outlining the study’s objectives was written and delivered to the administration of Pakistani FMCG consumers to request their desire to join in this study voluntarily. The confidentiality of company and employee identities was ensured. The endeavor was promised that no personally identifiable information would be used, published, or shared with a third party during the research. A total of 1,500 persons were contacted through email, and a survey was sent along with a covering note outlining the research’s goals and asking for their desire to cooperate. The investigators obtained permission from 1,000 employees to participate in this study voluntarily. The data collection procedure began on 12 December, 2021, and 600 completed questionnaires were collected on 3 February, 2022. As a result, with 67% of responses, the final response rate was 1,000.

### Measures of the Study

A 23-item questionnaire was devised to analyze the impulsive and compulsive buying tendencies and consumer resistance to digital innovations, and moderating role of the perceived threat of COVID-19 in the consumers of FMCG products from Pakistan.

1. A 5-item scale of impulsive buying was adopted by Kim and Seidlitz (2002) and Panagiotidou (2021). Items included in this scale are “I often buy things spontaneously” and “Sometimes I feel like buying things on the spur of the moment.” The results were collected by a “7-point Likert scale ranging from 1 = Strongly disagree to 7 = Strongly agree.”
2. A 3-item scale of impulsive buying was adopted by Japutra et al. (2019). Items included in this scale are, “My closet has unopened shopping bags of this brand in it” and “Others might consider me a shopaholic for this brand.” The results were collected by a “7-point Likert scale ranging from 1 = Strongly disagree to 7 = Strongly agree.”
3. A 7-item scale of consumer resistance to digital innovation was adopted by Hosseini et al. (2016). Items included in this scale are, “My closet has unopened shopping bags of this brand in it” and “Others might consider me a shopaholic for this brand.” The results were collected by a “7-point Likert scale ranging from 1 = Strongly disagree to 7 = Strongly agree.”
4. An 8-item scale of the perceived threat was adopted by Liu et al. (2021) and Paredes et al. (2021). Items included in this scale are “I believed that it was possible that I would contract COVID-19” and “The impact of the pandemic on people’s lives.” The results were collected by a “7-point Likert scale ranging from 1 = Strongly disagree to 7 = Strongly agree.”

## Results

### Measurement Model

#### PLS Algorithm

SmartPLS3 was used to assess the measurement and structural model. According to the simulation review, respondents’ gender and marital status substantially impacted their pleasure with and consumer resistance to digital innovation; hence, all two demographic variables were adjusted throughout the experiment. The detailed demographic statistics are mentioned in Table 1.

**TABLE 1:** Demographic profile.

Demography	Description	No. of responses	%
Gender	Male	780	78
	Female	220	22
Marital status	Married	650	65
	Not married	350	35

**FIGURE 2:**
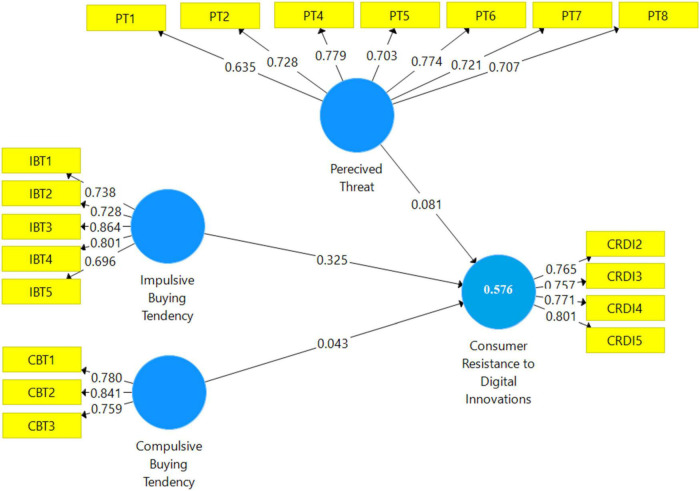
Measurement model.

As per the measurement model presented in Figure 2, additionally, “Cronbach’s alpha (CA)” and “composite reliability (CR)” values were computed using the measurement model to assess the coherence of the measurements (Raeder et al., 2008). All investigation items had “CA and CR values larger than 0.7,” indicating that they met the reliability criterion (Ramayah et al., 2018). Then, “factor loading” and “Average Variance Extracted” (AVE) values were calculated to determine the constructs’ convergent validity (Ramayah et al., 2018). In both studies, all factor loadings of the research constructs exceeded the minimal criteria of 0.70, and the AVE value was greater than 0.50 (Raeder et al., 2008). Similarly, all the above-discussed statistics for this study are presented in Table 2.

**TABLE 2:** Composite reliability, Cronbach’s alpha, and AVE values.

Constructs/Items	CA	Rho-A	CR	AVE
Compulsive buying tendency	0.707	0.715	0.835	0.627
Consumer resistance to digital innovations	0.777	0.781	0.856	0.599
Impulsive buying tendency	0.826	0.832	0.878	0.591
Perceived threat	0.829	0.834	0.875	0.540

*CR, composite reliability; AVE, average variance extracted; CA, Cronbach’s Alpha.*

Furthermore, the discriminant validity of all research models must be established. Fornell and Larcker (1981) described discriminant validity as “the extent to which a particular latent variable differs from other latent variables.” It was measured by analyzing the relationship between the latent variables item and the actual number of AVE (Raeder et al., 2008). Raeder et al. (2008), establishing discriminant validity, advised that latent factors with a value of 0.50 or above be used. According to Raeder et al. (2008), discriminant validity is shown whenever the square root of AVE is higher than the value of latent constructs. The discriminant validity statistics for this research are mentioned in Table 3.

**TABLE 3:** Discriminant validity.

	CBT	CRDI	IBT	PT
Compulsive buying tendency	0.792			
Consumer resistance to digital innovations	0.200	0.774		
Impulsive buying tendency	0.389	0.356	0.769	
Perceived threat	0.395	0.202	0.358	0.735

### Assessment of the Structural Model

#### Direct Hypothesis Testing

This section focuses on the structural model defined by Raeder et al. (2008) in terms of obvious measurement model relationships. The study’s postulated model employs a structural model to emphasize the relationship’s interdependence. In PLS, the structural model examines the direct relationship between the proposed hypotheses and their t-values and regression coefficients; according to Ramayah et al. (2018), an indirect effect is the same as a standardized beta value in regression analysis. The regression coefficient’s *t*-values and beta values are utilized to determine significance; according to Hair et al. (2017), *t*-values greater than “1.64” are considered statistically significant, and then used to make decisions on the proposed hypothesis. Examining direct linkages and verifying projected relationships between components using a structural model are the main goals of researching the model. This study investigates six hypotheses. SmartPLS 3.0 output findings, such as path coefficients, *t*-values, *p*-values, and standard errors, are provided in the table (Ramayah et al., 2018). The researcher used them to determine whether or not the hypothesis was supported. The direct hypothesis testing results are presented in Table 4.

**TABLE 4:** Hypothesis testing.

H	Path	*B*-value	Sample mean	Standard deviation	*T*-value	*P*-value	
H1	CBT > PT	0.297	0.297	0.072	4.119	0.000	Supported
H2	IBT > PT	0.219	0.223	0.076	2.891	0.004	Supported

*CBT, Compulsive Buying Tendency; PT, Perceived Threat; CRDI, Consumer Resistance to Digital Innovations; IBT, Impulsive Buying Tendency.*

In the above table, the results of compulsive buying tendency concerning the perceived threat accept the hypothesis. The results show that consumer resistance to digital innovations in relation to the perceived threat accepts the hypothesis. As the results show, impulsive buying tendency in relation to the perceived threat accepts the hypothesis.

#### Moderator Hypothesis Testing

An analysis of moderation was “used to discover which moderator variable changes the success or strength of the link between the independent and dependent variables,” according to Ramayah et al. (2018). The table shows that perceived threat (*B* = 0.083, *P* = 0.004) moderates the relationship between consumer resistance to digital innovations and impulse buying tendency, so this hypothesis is accepted. Perceived threat (*B* = 0.083, *P* = 0.004) moderates the relationship between consumer resistance to digital innovations and compulsive buying tendency, so this hypothesis is accepted. Detailed statistics for moderation results are presented in Table 5.

**TABLE 5:** Moderator hypothesis testing.

H	Path	*B*-value	(STDEV)	*T*-value	*P*-value	Decision
H3	CRDI * PI > IBT	0.083	0.045	3.485	0.004	Supported
H4	CRDI * PI > CBT	0.078	0.054	2.543	0.007	Supported

*p < 0.05

#### Assessment of R^2^

**FIGURE 3:**
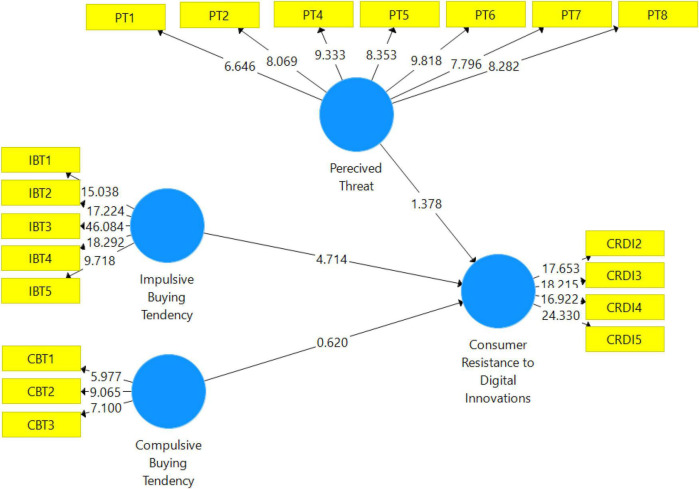
PLS bootstrapping.

The second stage in analyzing a structural model is determining the coefficient of determination (Hair et al., 2011). The variance in endogenous constructs caused by external constructs is represented by the coefficient of determination (Hair et al., 2011). The value of *R*^2^ ranges from zero to one. Moreover, Chin (1998) recommended that the *R*^2^ value of “0.13 is considered weak.” “0.33 is moderate,” and “0.67 is considered as strong.” The coefficient of determination for endogenous constructs is given in Table 6. PLS bootstrapping images and values are presented in Figure 3.

**TABLE 6:** Assessment of *R* square.

	*R* ^2^
Perceived threat	0.576

## Discussion

This study aimed to test the relationship between impulsive and compulsive buying behavior and consumer resistance to digital innovations. Additionally, this study examined the moderating role of perceived threat of COVID-19 on the relationship between impulsive and compulsive buying behavior and consumer resistance to digital innovations. The results support all hypotheses.

The existing study’s findings reveal a significant relationship between impulsive buying tendency, compulsive buying tendency, consumer resistance to digital innovation, and the moderating role of the perceived threat of COVID-19. Furthermore, demographic data suggested that males are more likely than females to consume FMCG products from Pakistan (Akram et al., 2022).

Additionally, the perceived threat of COVID-19 moderation has also proved the weakness and strength between impulsive, compulsive buying tendencies and consumer resistance to digital innovation.

Hypothesis 1 of this research suggested a positive association between impulsive buying tendencies and consumer resistance to digital innovations. The study revealed significant associations in results to support these proposed linkages. These findings were in line with the previous scholars (Panagiotidou, 2021). Similar patterns were also reported in a study by Japutra and Song (2020). Hypothesis 2 of this research proposed a positive association between compulsive buying tendencies and consumer resistance to digital innovation. Current research findings have supported this proposed relationship to prove that hypothesis 2 of this study held. Past research has also reported significant indirect results aligned with the findings of this research (Kaur et al., 2020; Tarka and Kukar-Kinney, 2022).

Similarly, hypothesis 3 of this research proposed that the perceived threat of COVID-19 moderates the relationship between impulsive buying tendency and consumer resistance to digital innovations in such a way that the relationship is weaker when the perceived threat of COVID-19 is high. The findings of this study proved this. So, hypothesis 3 of this research was approved. However, such a moderation study was not conducted earlier, and is a unique contribution of this research. However, it indirectly supports the causal associations recommended in the recent literature (Kim, 2020; Liu et al., 2021; Paredes et al., 2021). Hypothesis 4 of this research proposed moderating the influence of the perceived threat of COVID-19 between compulsive buying tendencies and consumer resistance to digital innovation. The study results revealed significant support for hypothesis 4 of this research. No preliminary evidence exists to confirm these results as a novel relationship was explored. However, these results are indirectly in line with some recent studies’ recommendations (Kaur et al., 2020; Kim, 2020).

## Conclusion

This study’s empirical findings describe the significance of relationships between impulsive buying tendency, compulsive buying tendency, consumer resistance to digital innovation, and the moderating role of the perceived Threat of COVID-19. Scholars and practitioners are becoming more interested in consumer resistance to digital innovation and compulsive and impulsive buying tendencies for FMCG produscts from Pakistan (Akram et al., 2022). Companies are obliged to overhaul their policies and rebuild their marketing strategies in the modern age to achieve impulsive and compulsive buying tendencies through consumer resistance to digital innovation. The key insights from this research build trust in business leaders that consumer resistance has a bright future and the involvement of management in implementing digital innovation in FMCG products from Pakistan. The research lays a solid platform for policy development and the creation of consumer resistance to digital innovation for several theoretical and practical insights into FMCG products.

### Theoretical Implications

The goal of this study was to make various theoretical advances in the field of knowledge. First, this study represents one of the earliest attempts to unite and integrate several tendencies into a unified theoretical framework. All of these traits have been investigated in various or distinct groups. Second, businesses might give purchasers regular notifications about their transactions, such as statuses, more photographs of the bought things, invitations to visualize oneself utilizing products online, and extra relevant details. It has contributed to the literature by bridging the theoretical gaps between consumer resistance to digital innovation, impulsive, compulsive buying behaviors, and the perceived threat of COVID-19. The study’s third important contribution was to suggest and test the mediation of the perceived threat of digital innovation between consumer resistance to digital innovation and tendencies. This expanded relationship study is a novel theoretical contribution that has provided empirical data to Pakistani FMCG product consumers. Lastly, this study helped unite two disparate theories of reasoned action and innovation resistance into a single paradigm. As an outcome of this combination, new pathways for future research in consumer resistance with theoretical implications have opened up.

### Practical Implications

The risk of high innovation failure rates is one of the biggest challenges that managers need to face. Our study provides policymakers, practitioners, and managers with useful insights. The results demonstrate that tendencies are the essential components in elaborating the idea of consumer resistance to digital innovation in determining the success of innovation in Pakistani FMCG products. Therefore, administrators and policymakers should look for criteria while implementing consumer resistance to digital innovation; impulsive and compulsive buying tendencies and perceived threats were the most critical determinants of the use of technology in consumers of FMCG products in Pakistan. The FMCG products’ entrepreneurs and managers may look for attributes such as impulsive and compulsive buying tendencies and the perceived threat of COVID-19 from consumer resistance to digital innovation. The findings suggest a link between consumer resistance to digital innovation and perceived threats in which the larger the perceived threat, the higher the customer resistance to change.

### Limitations and Future Research Directions

In addition to its many great aspects, the current study, like all others, has significant flaws that must be addressed in future research efforts. The current study was conducted among consumers of FMCG products from Pakistan related to the FMCG sector. Therefore, generalizing study results to other sectors may be an issue. Future research may include a diverse sample from various aspects of digital innovation and FMCG products in multiple businesses to reach generalizable results. Second, the obtained data were cross-sectional, despite the fact that future researchers may employ a longitudinal study design to determine causation with more accuracy. Researchers should consider variables that may mediate the effects to obtain more significant results in future studies because it is more common in industrialized countries to apply digital innovation for consumer resistance advancement. Finally, the full data collection process was conducted entirely online. As a result, consumers who did not have Internet access were eliminated. In future studies, data will be collected using a self-administered survey since most Pakistanis do not have Internet access.

## Data Availability Statement

The raw data supporting the conclusions of this article will be made available by the authors, without undue reservation.

## Author Contributions

HL had contributed in literature review part, while KH had contributed in writing introduction and theory part of this manuscript. HM and TK had contributed in methodology, data analysis, and results writing tasks. MA and MY have helped to improve the overall quality of article by reviewing and professional editing. All authors contributed to the article and approved the submitted version.

## Conflict of Interest

The authors declare that the research was conducted in the absence of any commercial or financial relationships that could be construed as a potential conflict of interest.

## Publisher’s Note

All claims expressed in this article are solely those of the authors and do not necessarily represent those of their affiliated organizations, or those of the publisher, the editors and the reviewers. Any product that may be evaluated in this article, or claim that may be made by its manufacturer, is not guaranteed or endorsed by the publisher.
